# What Will You Protect? Redefining Professionalism Through the Lens of Diverse Personal Identities

**DOI:** 10.15766/mep_2374-8265.11203

**Published:** 2021-12-02

**Authors:** Ananya Bhatia-Lin, Keith Wong, Rupinder Legha, Valencia P. Walker

**Affiliations:** 1 Second-Year Medical Student, University of California, Los Angeles, David Geffen School of Medicine; 2 Assistant Research Scientist, UCLA Center for Health Services and Society; 3 Associate Division Chief of Health Equity and Inclusion, Division of Neonatology, Department of Pediatrics, The Ohio State University College of Medicine; Associate Chief Diversity and Health Equity Officer, Nationwide Children's Hospital

**Keywords:** Professionalism, Professional Identity Formation, Narrative Medicine, Case-Based Learning, Diversity, Inclusion, Health Equity, Anti-racism

## Abstract

**Introduction:**

Professional identity formation (PIF) encapsulates the process of incorporating a physician's professional identity into existing personal identity. Medical schools shape PIF by reinforcing professional norms defined by a historical physician phenotype. Increasingly, medical students who are underrepresented in medicine must confront the apparent contradictions between personal identities and the often-subjective definitions of professionalism endorsed by faculty, patients, and peers. The lack of a framework for negotiating these conflicts can create barriers to achieving full academic and professional potential.

**Methods:**

We designed a 2-hour professionalism module during the first-year medical student orientation at one medical school. Participating students listened to a physician discuss a defining career moment that required reconciliation of personal and professional identities. Afterwards, students broke into small groups and discussed vignettes illustrating personal identities challenged by professionalism norms. Students then anonymously wrote a reflection about one aspect of their identity they intended to protect during their PIF process. An overwhelming majority of students posted their anonymous reflections on a wall for other students, staff, and faculty to view.

**Results:**

We analyzed the written reflective responses to the module. Several broad-ranging themes, including Mission, Identity, and Relationships, were identified. Both participant and facilitator evaluations were analyzed to determine the module's success.

**Discussion:**

This module provides a framework for faculty and administrators to create other curricular and pericurricular experiences that positively shape PIF. The session format utilized may generate greater interest in proactively supporting medical students as they navigate formation of their professional identities.

## Educational Objectives

By the end of this activity, learners will be able to:

1. Define professional identity formation, professionalism, and personal identity.
2. Summarize the complex factors (e.g., socioeconomic, racial, historical) that contribute to shaping the norms of the medical profession.
3. Recognize situations where medical students may experience challenges to their personal identity due to deviations from established professionalism norms in medical education.
4. Develop individual tools to address conflicts between professionalism and personal identity in order to prevent maladaptive professional identity formation.

## Introduction

Becoming a physician involves profound personal transformation from preprofessional identity to an identity as a professional physician. Known as professional identity formation (PIF), this dynamic process relies on implicit and explicit lessons provided within medical education.^1–3^ Physician PIF has historically been racialized and gendered to reflect the White male physicians who dominated the occupation and to maintain an overrepresentation among its leadership. Ongoing systemic biases, institutional racism, and other structural factors limiting the entry of non-White populations into medicine uphold this racialized norm of the physician professional.^4,5^ Medical students and trainees face prejudiced behavior from patients, peers, educators, and mentors within the medical institution.^5,6^ As a protective strategy, students underrepresented in medicine (URiM) may hide, or cover, the stigmatized identities in conflict with this norm. However, these behaviors can trigger emotional and social stresses that derive from the created dissonance between their actual professional identity and the stereotypically constructed ideals of professionalism within medicine.^7,8^ Without the skills to navigate identity maintenance during PIF, students, especially those who are URiM, may suffer from an impaired sense of self, increased stress, and mistreatment leading to burnout.

Recent publications on professional behavior in medicine have invigorated discussions about the burden of racialized and gendered professionalism norms on increasingly diverse medical students and trainees. Narrative evidence from URiM professionals reveals they repeatedly face racialized and/or gendered microaggressions that they often feel compelled to ignore in order to maintain professionalism.^6,8–10^ These experiences unfairly burden URiM physicians and adversely affect their health and safety. The increasingly recognized harm caused by these outdated professionalism norms calls into question the appropriateness of teaching and enforcing them within medical education. New curricula on professionalism characterize PIF as an individual and highly variable process that builds off one's preexisting social identity.^11,12^ However, no existing PIF curricula sufficiently address the rigidity of professionalism norms and their clash with individuals from marginalized and historically excluded identities. We sought to build a module that would address strategies for expanding professionalism norms to better incorporate diverse racial identities, genders, sexualities, and other marginalized markers of identity. We took inspiration from student-led modules on bias, stereotyping, and racism in medicine to redesign our didactic session as a small-group, learner-focused experience.^13,14^

Previously at our single-site medical school, professionalism was taught through didactic sessions listing expectations and case examples of unprofessional behavior (Appendix A). In recent years, faculty responsible for these presentations faced challenges with addressing a growing number of concerns raised by students during these sessions. Students questioned seemingly illogical nuances to descriptions of professional appearance and dress code violations and proposed reform of established professionalism norms that created harm for students with personal identities that differed from the historic White, cisgendered male model of the medical professional. Lack of additional instructional opportunities on professionalism further exacerbated these issues. To this end, we proposed an innovative educational approach designed to minimize the harm caused by covering expressions of personal identity and to foster a process of PIF within our institution that would integrate students' values and personal backgrounds.

## Methods

For the innovation, we replaced the initial didactic lecture on professionalism (Appendix A) with one didactic session and one small-group session (Figure). The prior learning objectives were covered in the didactic session, while the small-group session was designed to address new educational objectives. Facilitators (senior students, staff, faculty, assistant deans) were chosen from the Office of Equity, Diversity, and Inclusion, the Student Affairs Office, and the Academic Support Office. These facilitators were provided with a facilitator guide and a de-escalation plan in the event of interpersonal conflict. Facilitators were instructed to allow for participant-directed learning and encouraged to refrain from adding their personal contributions to discussions. They were also prompted to alert the assistant dean of students assigned to each group if participant discussion required de-escalation.

**Figure.**
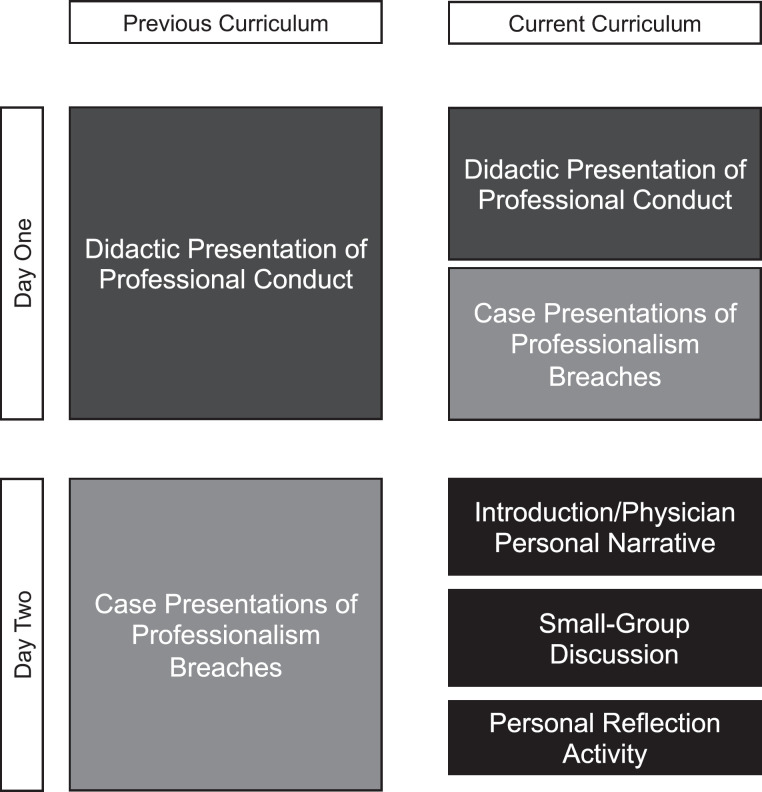
Design of the Transition to the Profession curriculum as it compares to the previous curriculum.

In the 2019 module, 181 first-year medical students participated. The didactic session contained core tenets of professionalism as well as information about the administrative consequences of breaches of professionalism. Small-group sessions began with prereadings on the interaction between personal identity and professional norms (Appendix B).^6^ A faculty member then shared an experience of dealing with prejudicial behavior from patients. Afterwards, within their small groups, students read and discussed vignettes drawn from common medical student experiences (Appendix C). Facilitators moved among small groups to monitor discussions and de-escalate any conflicts. No incidents requiring de-escalation occurred.

The selected prereadings and shared physician narrative provided examples of personal identity intersecting with professionalism in medicine. One reading described how elements of personal expression that conveyed the importance of shared community identity (e.g., earrings) were deemed unprofessional by supervising attendings. Another reading illustrated the experiences of a Black woman in medicine during a time where her personal suffering from the harms of anti-Black racism was also characterized as unprofessional. The physician narrative chosen for this module described a Muslim woman physician facing prejudicial behavior from a patient and highlighted the impact on her sense of self as a physician. The physician narrative also included details as to how the physician and a member of the physician's team addressed the situation. The intention of including a physician's personal experience was for all participants to experience a compelling narrative defining a perceived conflict between personal identity and professional identity. The emotional impact of this conflict was described, as well as the impact of the physician's own actions and the actions of the physician's peers.

At the end of the session, students reflected on the question “What [of your premedical identity] will you keep as you transition to medicine?” They wrote their responses anonymously on adhesive-backed notes and publicly posted their answers. Designed to highlight the aspects of personal identity defined as most essential to maintain during PIF process, the question placed no restrictions or guidelines on how students could respond. Students were free to use multiple adhesive-backed notes to answer. While introducing students to challenges they or their peers might face as they began the PIF process, the session also created moments to positively affirm the unique elements of student identities. Additionally, it afforded students an opportunity to construct responses for how they might respond in the event they experienced microaggressions in their professional environments.

The activity generated 249 narrative notes to transcribe and analyze using content analysis.^15^ Key concepts from the text were coded into discrete categories by theme and subtheme. Two independent coders performed data reconciliation. Discrepancies in coding were discussed and reconciled. Narrative from each note could be coded as multiple themes or subthemes based upon content. When the narrative from a note explicitly stated a theme (e.g., Identity), no additional subcategorization was performed. The purpose of analyzing these free-form notes was to discover what elements of their personal identities students felt strongly about protecting during their transition to a professional physician identity.

The next iteration of this module changed to a virtual format due to the COVID-19 pandemic. In adjusting to the virtual platform, an introductory slideshow was prepared to define PIF and describe challenges students might face in their processes of PIF (Appendix D). Facilitators were provided with a facilitator guide delineating the role of the facilitator as well as guidelines for addressing potentially problematic behavior or escalations in the discussion (Appendix E). Additional revisions included the incorporation of a new reading addressing experiences with professionalism as a Black physician.^7^ The faculty narrative was shared with a large group of all 180 students via an online platform, and then students transitioned into 10-person breakout rooms. Although virtually present, facilitators kept their cameras and microphones off within the student breakout rooms, and this expectation of facilitator silence was shared with student participants. No discussions or disagreements required de-escalation. A digital survey tool was used to collect anonymous student answers to the “What will you keep?” question and student feedback on the session (Appendix F). Facilitator feedback was also solicited (Appendix G).

In both iterations of the workshop, students received a didactic presentation on PIF and professionalism norms, as well as examples of personal identity conflicts with historical norms of professionalism. Students were then expected to guide their own small-group discussions using the vignettes for structure. Students drew from personal experience as well as knowledge of current and historical events to explain challenges and opportunities within PIF to themselves and their peers. Facilitator and student feedback, including individual reflections on their experiences during these sessions, was solicited.

## Results

Several major themes developed during review of the anonymous narratives provided by students: Mission referred to the students' ambitions for their future in medicine. Identity encapsulated intrinsic and extrinsic factors about the student. Relationships represented the final major theme identified in the analysis. Due to the breadth of responses, Identity and Relationships underwent additional subcategorization. (Table 1 gives a full list and examples of the themes and subthemes recognized.)

**Table 1.**
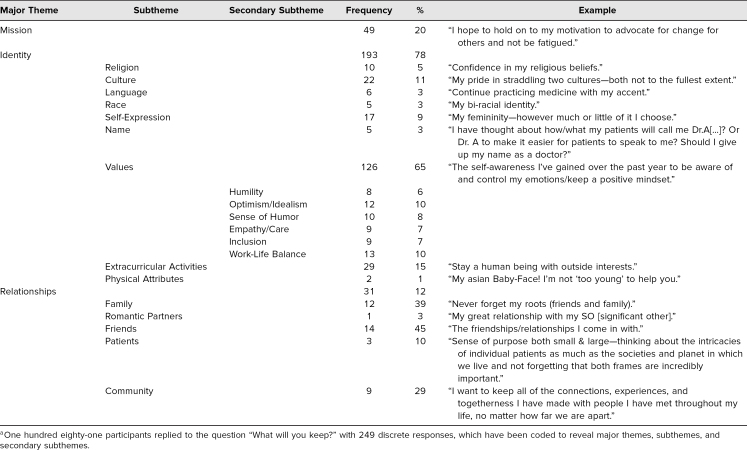
Results of Personal Reflection Activity During First-Year Student Orientation, August 2019^a^

Identity subthemes included Religion; Culture, as it pertained to the family background of students; Language; and Race. Self-Expression, or the outward style and expression students chose to display, and Name fit into the Identity subthemes. Values, including morals as well as what students valued about their intrinsic selves, were a prominent subtheme as secondary subthemes emerged from them, such as Humility, Optimism/Idealism, Sense of Humor, and Work-Life Balance. Extracurricular Activities, or activities and hobbies that students did outside of school, and Physical Attributes, or immutable elements of students' appearance, were strongly associated subthemes of Identity. The Relationships theme was subcategorized based upon the nature of the interaction with the student. Its common subthemes included Family, Friends, Romantic Partners, Patients, and Community. Community appeared to describe relationships with groups of people.

Students remained highly engaged during the sessions, irrespective of in-person or digital learning format. In participant evaluations, students indicated that (1) these topics were relevant to their medical school experience, (2) the experiences described within the activity were realistic, and (3) the module as designed allowed them to safely share their opinions (for more details, see Table 2).

**Table 2.**
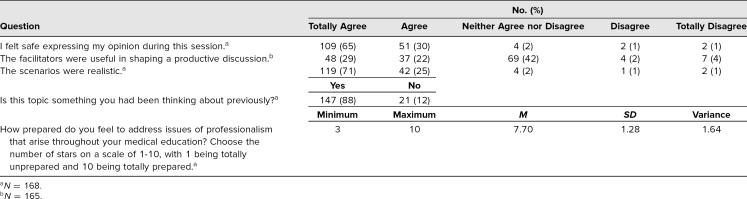
Survey Data From Participants Evaluating Their Experience During the Virtual Session

Additional information to evaluate the success and feasibility of this innovation included review of facilitator feedback obtained via open-ended survey questions. All staff, faculty, and student facilitators were sent online surveys with the following three questions:

• “What went well?”
• “Please elaborate on any issues that occurred during the session. For instance, if a student said something inappropriate or another student appeared negatively impacted by something someone said.”
• “Any areas of concern to consider in future?”

These surveys were completed by all participating facilitators with a 100% response rate. Facilitator reports highlighted that the diversity of student experience translated to rich and meaningful conversations on expression of identity and professionalism. In describing the experience, one facilitator reported,

The students were all very open and respectful, and shared their personal experiences. I think that the depth of honesty about their lived experiences was very powerful in bringing them closer together. They shared current thoughts about balancing being who they are in contrast to perceived expectations for how a doctor should be.

## Discussion

The impetus for creating this module came from our medical student authors recognizing how their peers struggled with exclusionary norms that seemed to define a professional physician. Together, our goal was to build the groundwork to center and celebrate personal, preprofessional values, ambitions, relationships, and markers of cultural identity throughout individual processes of PIF. We aimed to foster empathy for others' personal experiences, demonstrate how personal identity and professionalism can conflict, and prime students to support each other through these conflicts as they become professional physicians.

Answers to the provocative question “What will you keep?” inform some of the most salient elements of personal identity as students prepare to enter the medical school environment. These elements of cultural background, personal values, future dreams, and the ability to sustain preferred relationships and activities often suffer from detrimental deprioritization during a maladaptive PIF process. This negatively affects mental and physical health as well as future professional career advancement. In contrast, the themes and subthemes identified by this innovation may inform medical education leaders of creative ways to reimagine medical education and decrease burnout among students and trainees. In our evaluation of the student reflections submitted, there was a large breadth of salient personal identity factors, as expected from a diverse student body. Prominent among these were features such as multilingual ability, physiognomic characteristics, culturally significant body piercings, and other aspects of expressed identity that challenged the White, cisgendered, able-bodied norm of the professional physician.

Our module implementation, however, also faced some limitations. First, our evaluations were not designed to assess downstream effects such as decreased burnout, improved student performance, and enhanced sense of belonging at our institution. Second, because this was a student-led module, we lacked a defined pathway to implement results obtained from the module into the remainder of the curriculum. Likewise, the module was not designed to initiate institutional changes to the overall learning environment.

The module remains a core element of the medical school's first-year orientation curriculum. Opportunities to build out our session content include adding reflection exercises on PIF at subsequent time points within the medical school curriculum. We think this would provide particular value for students as they attain clinical experiences and skills that further shape their identity as physicians. Many facilitators suggested creating a longitudinal curriculum on PIF at our institution. In addition, individual students expressed appreciation for the opportunity to discuss these topics at the very beginning of their matriculation and requested more engagement with this content throughout their medical school education. The development process and early experience of this intervention may provide a model for other medical schools to incorporate explicit teaching about professionalism upon entry of students into medical school.

By defining essential elements of their personal identity and anchoring them within the PIF process, students can potentially construct precision road maps that optimize their learning. Medical education leaders might also use aggregations of these road maps as an innovative method for transforming their approach to revising medical school curricula. Moreover, recognition of these essential identity traits ought to trigger proactive protection plans that prevent suppression or extinction of positive and important personal characteristics when students experience biased or prejudiced behaviors from others during the course of their medical training. This leaves at least one major question for medical education leaders and experts to consider. How might policies, programs, and experiences involved in PIF not only protect but also affirm and celebrate the inherently unique and invaluable traits of medical students? The glaring need for more respectful and compassionate physicians who practice just and equity-driven clinical care demands a pursuit of answers to that question.

## Appendices


Prior Professionalism Lecture.pptTransition to the Profession Prereadings.docxTransition to the Profession Vignettes.docxTransition to the Profession.pptFacilitator Guide.docxTransition to the Profession Student Feedback.docxTransition to the Profession Facilitator Feedback.docx
*All appendices are peer reviewed as integral parts of the Original Publication.*
