# Supplementary material for: What Will You Protect? Redefining Professionalism Through the Lens of Diverse Personal Identities
Source: MedEdPORTAL. 2021 Dec 2;17:11203. doi: 10.15766/mep_2374-8265.11203 (PMC8636300; doi:10.15766/mep_2374-8265.11203)
Supplement: Supplementary file 1 — Prior Professionalism Lecture.pptTransition to the Profession Prereadings.docxTransition to the Profession Vignettes.docxTransition to the Profession.pptFacilitator Guide.docxTransition to the Profession Student Feedback.docxTransition to the Profession Facilitator Feedback.docx [file mep_2374-8265.11203-s001.zip › C. Transition to the Profession Vignettes.docx]

Transition to the Profession

DGSOM MS1 Orientation 2020

Vignettes for table top discussions (Small Groups)

1. It is your first day of medical school. You and your classmates are meeting with your small group tutor. Your tutor asks for introductions, and when you say your name the tutor asks that you repeat it a couple of times. Finally, the tutor says, “Well I have never heard of that name before, I can’t really pronounce it, so I am just going to call you ‘Joe’.” You are surprised but not sure how to respond. You look around the group, but everyone is quiet as they move onto the next student.
   1. How does this make you feel?
   2. What does your name mean to you?
   3. What do you want to say? Why would this be difficult?
   4. You are a classmate in the small group- what would you like to say?
2. You are getting ready for your very first clinical clerkship – exciting! In with the new earrings – and oh those new shoes look sharp! And now, which hijab to pair with the new outfit?? Hmm, you hesitate – a hijab. Should I wear any hijab?? What will by patients think? My Attending? What might they say to me – and how will I respond?

Additional questions for discussion:

1. What were the choices that the students had to make in these two scenarios? Why do you think they had to make these choices? What were their options? What were the risks and benefits of each option?
2. If you were in their place, what decisions would you make? Why?

Personal reflection:

1. Reflect on your own life. What sort of decisions about your identity expression do you think you will need to make as you navigate the medical profession?
