# Supplementary material for: What Will You Protect? Redefining Professionalism Through the Lens of Diverse Personal Identities
Source: MedEdPORTAL. 2021 Dec 2;17:11203. doi: 10.15766/mep_2374-8265.11203 (PMC8636300; doi:10.15766/mep_2374-8265.11203)
Supplement: Supplementary file 1 — Prior Professionalism Lecture.pptTransition to the Profession Prereadings.docxTransition to the Profession Vignettes.docxTransition to the Profession.pptFacilitator Guide.docxTransition to the Profession Student Feedback.docxTransition to the Profession Facilitator Feedback.docx [file mep_2374-8265.11203-s001.zip › F. Transition to the Profession Student Feedback.docx]

Transition to the Profession

Student Feedback Form

Q1. What elements of your pre-medical identity do you want to keep with you as you transition to the medical profession?

Q2. What challenges do you foresee in maintaining your personal identity?

Q3. What steps will you take to address these challenges?

Q4-6. On a scale from Totally Agree to Totally Disagree.

| # | FIELD | Totally Agree | Agree | Neither Agree Nor Disagree | Disagree | Totally Disagree |
| --- | --- | --- | --- | --- | --- | --- |
| Q4 | I felt safe expressing my opinion during this session |  |  |  |  |  |
| Q5 | The facilitators were useful in shaping a productive discussion |  |  |  |  |  |
| Q6 | These scenarios were realistic |  |  |  |  |  |

Q7. Is this topic something that you were thinking about previously?

___YES ____NO

Q8. How prepared do you feel to address issues of professionalism that arise throughout your medical education?

Choose on a scale from 1-10, with 1 being totally unprepared and 10 being totally prepared.

| 1 | 2 | 3 | 4 | 5 | 6 | 7 | 8 | 9 | 10 |
| --- | --- | --- | --- | --- | --- | --- | --- | --- | --- |
| Totally unprepared |  |  |  |  |  |  |  |  | Totally prepared |

Q9. If you have any comments or questions, please share them below.
