# Supplementary material for: What Will You Protect? Redefining Professionalism Through the Lens of Diverse Personal Identities
Source: MedEdPORTAL. 2021 Dec 2;17:11203. doi: 10.15766/mep_2374-8265.11203 (PMC8636300; doi:10.15766/mep_2374-8265.11203)

Transition to the Profession

Facilitator Feedback & Session Debrief

Hello and thank you for your participation in the “Transitioning to the Profession” Orientation session! The organizers express their gratitude for your contributions to this event. Please fill out the form below with your reflections and feedback.

Please write your name on this form if you'd like us to follow up with you regarding one of your comments.

1. Which session did you participate in/facilitate?
2. General structure of session (what was the "flow?")
3. What went well?
4. If there were any issues in your sessions, please elaborate. For instance, if a student said something inappropriate or another student appeared negatively impacted by something someone said.
5. What would you improve on?
6. Any areas of concern to consider in future?


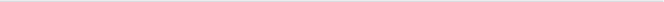

Supplement: Supplementary file 1 — Prior Professionalism Lecture.pptTransition to the Profession Prereadings.docxTransition to the Profession Vignettes.docxTransition to the Profession.pptFacilitator Guide.docxTransition to the Profession Student Feedback.docxTransition to the Profession Facilitator Feedback.docx [file mep_2374-8265.11203-s001.zip › G. Transition to the Profession Facilitator Feedback.docx]
