# Supplementary material for: What Will You Protect? Redefining Professionalism Through the Lens of Diverse Personal Identities
Source: MedEdPORTAL. 2021 Dec 2;17:11203. doi: 10.15766/mep_2374-8265.11203 (PMC8636300; doi:10.15766/mep_2374-8265.11203)
Supplement: Supplementary file 1 — Prior Professionalism Lecture.pptTransition to the Profession Prereadings.docxTransition to the Profession Vignettes.docxTransition to the Profession.pptFacilitator Guide.docxTransition to the Profession Student Feedback.docxTransition to the Profession Facilitator Feedback.docx [file mep_2374-8265.11203-s001.zip › B. Transition to the Profession Prereadings.docx]

Transition to the Profession

Pre-Readings:

| TITLE | HYPERLINK | CITATION |
| --- | --- | --- |
| “Diversity and Inclusion in Medical Schools: The Reality” | <https://blogs.scientificamerican.com/voices/diversity-and-inclusion-in-medical-schools-the-reality/> | Tsai J. Diversity and Inclusion in Medical Schools: The Reality. Scientific American Blog Network. Accessed October 28, 2019.<https://blogs.scientificamerican.com/voices/diversity-and-inclusion-in-medical-schools-the-reality/> |
| “Professionalism 101 for Black Physicians” | <https://www.nejm.org/doi/10.1056/NEJMpv2022773> | AbdelHameid D. Professionalism 101 for Black Physicians. New England Journal of Medicine. 2020;383(5):e34. doi:[10.1056/NEJMpv2022773](https://doi.org/10.1056/NEJMpv2022773) |
