# Supplementary material for: What Will You Protect? Redefining Professionalism Through the Lens of Diverse Personal Identities
Source: MedEdPORTAL. 2021 Dec 2;17:11203. doi: 10.15766/mep_2374-8265.11203 (PMC8636300; doi:10.15766/mep_2374-8265.11203)
Supplement: Supplementary file 1 — Prior Professionalism Lecture.pptTransition to the Profession Prereadings.docxTransition to the Profession Vignettes.docxTransition to the Profession.pptFacilitator Guide.docxTransition to the Profession Student Feedback.docxTransition to the Profession Facilitator Feedback.docx [file mep_2374-8265.11203-s001.zip › E. Facilitator Guide.docx]

Transitioning to the Profession

Facilitator’s Guide:

**Purpose**: The “Transitioning to the Profession” session was designed to (1) introduce students to the process of Professional Identity Formation and (2) give students the tools to address contradictions between “professionalism” norms and personal identity.

**Personnel needed:**

1. Course director: Will monitor main Zoom session
2. Facilitators (multiple): 1-2 facilitators per small group/breakout room of 6-10 students

**Advance Preparation:**

In advance, establish a notification system should a facilitator require assistance during a breakout room discussion. For instance, you can leave the breakout room and return to the main Zoom room to notify the course director. Another option would be to acquire the course director’s email or phone number prior to the session to contact them directly.

**Facilitator Role:**

As a Facilitator, your role is to support students as they discuss the vignettes provided and address the discussion questions as a group. All students should feel safe to share their opinion during this session.

**Schedule/Timing**

| **TIMING** | **ITEM** | **LOCATION** | **REFERENCE** | **COURSE DIRECTOR ROLE** | **FACILITATOR ROLE** |
| --- | --- | --- | --- | --- | --- |
| 00:00-00:10 | Didactic Presentation | Large Groups | Appendix D: Transition to the Profession | Present didactic presentation on PIF | Listen to large group presentation |
| 00:10-00:15 | Physician Personal Narrative | Large Groups | Appendix D: Transition to the Profession | Introduce speaker/prerecorded video (if applicable.) | Listen to large group presentation |
| 00:15-00:18 | Transition to Small Groups | Large Groups | Appendix D: Transition to the Profession | Introduce small group activity and individual reflections.  Describe intent of activity and set group intentions for the space. | Listen to large group presentation |
| 00:18-00:53 | Small Group Discussions | Small Groups/ Breakout Rooms | Appendix C: Transition to the Profession Vignettes  Appendix F: Transition to the Profession Student Feedback | Remain in main room (zoom or physical location) or be “on call” to troubleshoot student issues or technical difficulties | Stay in a small group/breakout room. Passively listen to students as they discuss the vignettes and PIF.  If there is an issue requiring facilitator intervention, please refer to facilitator guidelines for next steps. |
| -- | Post-Session Feedback | -- | Appendix G. Transition to the Profession Facilitator Feedback | -- | Fill out facilitator feedback form |

**Guidelines for Small Group Discussions:**

- Allow for self-directed learning
  - Zoom name = “Your Name (Facilitator”)
  - Facilitator will keep their video off and remain muted if they are not needed to maintain psychological safety for participants (see next point)
  - Inform students that they are expected to lead the entire discussion amongst themselves, including determining the timing of the discussion.
  - Allow for silence (Try not to ask questions or redirect the conversation if there has been less than 30 seconds of silence.)
- Maintain psychological safety for all participants
  - At the start of the session, provide students with the option to leave the breakout room and return to the main Zoom room if they need to take a break from the discussion
  - If someone says something potentially hurtful, remind people of the intention set at the beginning of the session

*“The intention of this session is to listen to each other, learn from each other, and support one another in the process of Professional Identity Formation”*

- - If a situation escalates, use the agreed upon method(s) to send a notification requesting assistance.
  - Take note if you see a student has been impacted by the session (try to note name) for follow-up by the course director.
- Make things better:
  - Please fill out a Facilitator Feedback form afterward and document what happened during your session as well as suggestions for improvement
